# Effect of magnetic field strength and segmentation variability on the reproducibility and repeatability of radiomic texture features in cardiovascular magnetic resonance parametric mapping

**DOI:** 10.1007/s10554-024-03312-7

**Published:** 2025-01-08

**Authors:** Pascal Yamlome, Jennifer H. Jordan

**Affiliations:** 1 Department of Biomedical Engineering, College of Engineering, Virginia Commonwealth University, Richmond, VA USA; 2 Division of Cardiology, Pauley Heart Center at Virginia Commonwealth University, Richmond, VA USA

**Keywords:** Radiomics, Reproducibility, Repeatability, Cardiovascular magnetic resonance imaging, Parametric maps

## Abstract

Our study aims to assess the robustness of myocardial radiomic texture features (RTF) to segmentation variability and variations across scanners with different field strengths, addressing concerns about reliability in clinical practices. We conducted a retrospective analysis on 45 pairs of CMR T1 maps from 15 healthy volunteers using 1.5 T and 3 T Siemens scanners. Manual left ventricular myocardium segmentation and a deep learning-based model with Monte Carlo Dropout generated masks with different levels of variability and 1023 RTFs extracted from each region of interest (ROI). Reproducibility: the extent to which RTFs extracted from 1.5 T and 3 T images are consistent, and repeatability: the extent to which RTFs extracted from multiple segmentation runs at the same field strength agree with each other, were measured by the intraclass correlation coefficient (ICC). We categorized ICC values as excellent, good, moderate, and poor. We reported the proportion of RTFs that fell in each category. The proportion of RTFs with excellent repeatability decreased as the proportion of ROI pixels in congruence across segmentation runs decreased. Up to 31% of RTFs showed excellent repeatability, while 35% showed good repeatability across segmentation runs from the manually generated masks. Across scanners (i.e., 1.5 T vs 3 T), only 7% exhibited good reproducibility. While our results demonstrate RTF sensitivity to differences in field strength and segmentation variability, we identified certain preprocessing filters and feature classes that are less sensitive to these variations and, as such, may be good candidates for imaging biomarkers or building machine-learning models.

**Supplementary Information:**

The online version contains supplementary material available at 10.1007/s10554-024-03312-7.

## Introduction

Radiomic texture feature (RTF) analysis is gaining traction as a valuable tool in medical image analysis with applications in computed tomography (CT) and magnetic resonance image examinations to extract higher-order quantitative information such as contrast, homogeneity, correlation, entropy, and pixel nonuniformity [1]. RTF analysis helps evaluate image textures and gives useful information about medical conditions, including tumors that might otherwise not be noticed, with standard quantitative image analyses based on the average and variation of voxel values. In the field of cardiovascular magnetic resonance (CMR) imaging, texture feature analysis of parametric maps has the potential to quantify and differentiate between the various types of fibrosis where the average T1 is non-discriminating. As such, RTFs have been used to assess myocardial infarction and differentiate between hypertensive heart disease and hypertrophic cardiomyopathy (HCM) [2, 3].

While RTFs offer promising capabilities for faster and more accurate diagnosis, their full integration into clinical practices is approached with caution due to the need for a deeper understanding of how radiomic workflow variations might impact the reliability of these features. It is widely recognized that variations can be introduced at multiple stages in the radiomic workflow, including image acquisition, segmentation, and feature extraction [4, 5]. Such variations may stem from factors such as scanner settings, imaging protocols, and human error during segmentation or feature extraction. As a result, there has been a significant effort to investigate and address these sources of variation, aiming to improve the reproducibility and repeatability of radiomic analyses. One such effort is the Image Biomarker Standardization Initiative (IBSI), which seeks to establish a set of guidelines and metrics for radiomic feature extraction [6]. Most of these efforts, however, have been directed toward CT and other modalities [7]. In the cardiovascular magnetic resonance imaging field, where tissue characterization is increasingly becoming important, the effects of variations in the radiomic workflow on extracted features remain largely unexplored [8–11].

A limited number of studies have explored the reliability of RTFs in CMR imaging [12]. These studies have investigated the effect of image resampling and discretization on the estimate of myocardial RTFs from T1 and T2 mapping in hypertrophic cardiomyopathy [13], studied the sensitivity of RTFs from varying sequence parameters on 3 T [14, 15], and studied the repeatability of RTFs in a multicenter, multi-vendor test–retest study [16]. While these studies have reported a wide range of repeatability in CMR radiomic features, they encouraged further investigations to elucidate the effects of variations in the radiomic pipeline on the reliability of RTFs. To date, the impact of common variations, such as changes in the captured region of interest and differences in magnetic field strength, on the repeatability and reproducibility of radiomic texture features remains largely unexamined. Understanding the robustness of RTFs to expected changes in the imaging/processing pipeline becomes particularly important for researchers conducting extensive multicenter studies with different imaging systems.

Additionally, segmentation variability, a widely acknowledged but insufficiently explored factor in the radiomic workflow, introduces a notable source of variation. Recent breakthroughs in deep learning and the introduction of Monte Carlo dropout (MCD), a technique for quantifying uncertainty in neural networks, allow us to generate diverse yet valid segmentation masks with varying degrees of variability. This presents a unique opportunity to comprehensively evaluate the impact of various levels of segmentation variability on myocardial RTF repeatability. Against this background, the present study sought to investigate the impact of magnetic field strength and segmentation variability on the reproducibility and repeatability of RTFs in CMR images in a healthy cohort. Specifically, we aim to assess (1) the effects of segmentation variability on the repeatability of CMR RTFs and (2) the reproducibility of RTFs across scanners with different magnetic field strengths (3 T vs 1.5 T).

This study enhances the current understanding of RTF applications in CMR by making the following contributions. First, we identify subsets of RTFs, feature classes, and image preprocessing filter types that demonstrate robustness to small changes in segmentation masks. This analysis addresses a key concern in radiomics by pinpointing features that remain stable despite inevitable variations in segmentation, a step that often introduces variability into the radiomic workflow. Furthermore, we identify RTFs, feature classes, and preprocessing filters that produce consistent results across scanners with different magnetic field strengths, specifically between the 1.5 T Siemens AREA and the 3 T VIDA scanners. By evaluating the impact of segmentation variability and magnetic field strength on the reproducibility and repeatability of RTFs, this study provides novel, valuable insights into the reliability of RTFs as imaging biomarkers for cardiovascular diseases. These findings will contribute to a better understanding of radiomics in CMR imaging and can help streamline future clinical or research radiomics-based machine learning pipelines by ensuring that only reproducible and repeatable RTFs are extracted.

## Methods

### Study population

**Fig. 1:**
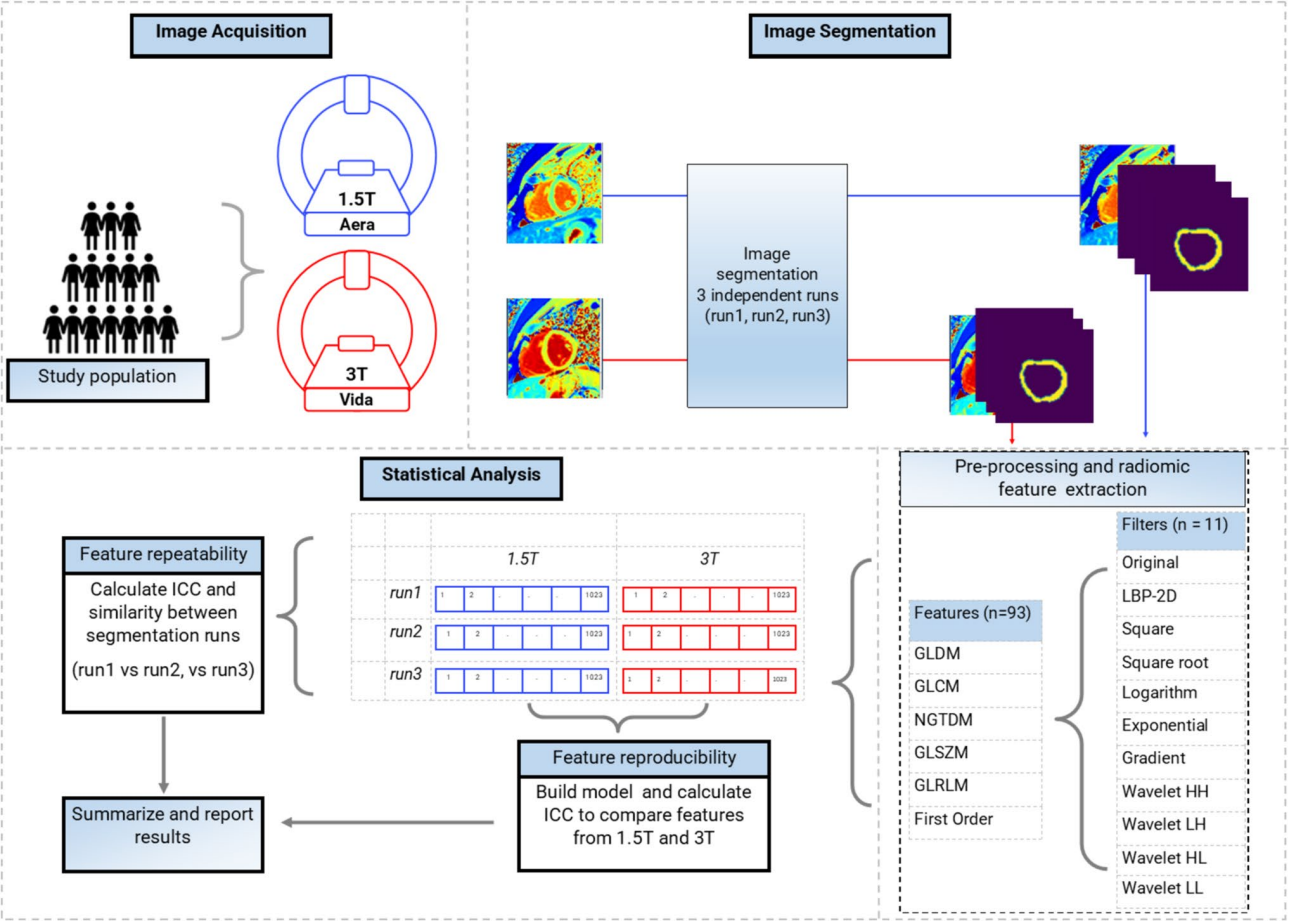
This flowchart shows the study pipeline from image acquisition, segmentation, and feature extraction to the analysis of extracted features. Three image segmentation runs (run1, run2, run3) were performed blind to prior segmentations with each performed one week after the previous segmentation run. From each segmented LV myocardium, we preprocessed the image with ten filters and extracted 93 features from the 11 versions of the image (the original image and ten filtered; 1023 features in all). The features can be grouped into six feature classes

We conducted a retrospective analysis on 45 paired CMR scans from 15 healthy volunteers (7 male and 8 female, age 41 ± 7 years), Fig. 1. Each volunteer underwent imaging with short-axis T1 maps acquired at the base, mid, and apex using both 1.5 T and 3 T scanners, resulting in a total of 90 images for analysis. Deidentified scans were performed in 2019 on healthy volunteers to collect magnet-specific normative data on T1 means and standard deviations in compliance with the Society of Cardiovascular Magnetic Resonance (SCMR) Imaging recommendations for parametric mapping [17]. The paired scans were performed within the same hour on each volunteer on a Siemens 1.5 T Aera and a Siemens 3 T MAGNETOM Vida. The present study focused on extracting and evaluating RTFs for robustness. Inclusion criteria for the study included being in good health and having no contraindications for CMR. Exclusion criteria included previous medical conditions or medications that may affect the imaging results. The present study was designated as non-human subjects research by the institutional IRB, given no identifying information was saved during the collection of the normative scans.

### T1 images

For each field strength, i.e., 1.5 T and 3 T, three short-axis T1 maps were acquired, one each at the basal, mid, and apical cavity. Eight T1-weighted images were acquired using the Siemens MyoMaps MOLLI 5(3)3 T1 sequence. The imaging parameters included a slice thickness of 8 mm, a flip angle of 35°, and an echo train length of 1. Additional magnet-specific technical details are as follows: for the 1.5 T images, the field of view (FOV) was 306 × 360 cm, the percent phase FOV was 85.16%, the echo time (TE) was 1 ms, the repetition time (TR) was 314.20 ms, and the acquisition matrix size was 256 × 218. For the 3 T images, the FOV ranged from 278–306 × 340–360 cm, the percent phase FOV varied from 83.59% to 85.95%, the echo time (TE) was 1.1 ms, the repetition time (TR) ranged from 287.60 ms to 322 ms, and the acquisition matrix size was between 131–256 × 131–146. Detailed magnet and scan-specific acquisition parameters can be found in Online Resource 1 (Table S1). Breath-holding and prospective ECG gating were employed to synchronize image acquisition with the cardiac cycle to minimize motion artifacts. The eight T1-weighted images were corrected for motion inline, and a three-parameter curve fitting was performed to generate the T1 map.

### Myocardial segmentation

A single analyst performed three blinded segmentations (run1, run2, and run3) of each map using custom MATLAB-based software tailored for CMR analysis [18], each run conducted a week apart. To assess the concordance of the Region of Interest (ROIs) obtained from the three segmentation runs, we adapted the concept of Intersection over Union (IoU) [19, 20] to measure the proportion of area captured in the three segmentation masks A_1_, A_2_, and A_3_ that achieved consensus between all three (IoU_3_), any two (IoU_2_), and only one segmentation run (IoU_1_). These coefficients were used to measure the proportion of any foreground/ROI pixel captured by all segmentations that achieved congruence between all three segmentations (Eq. 1), any two segmentations (Eq. 2), and no agreement (Eq. 3).

1 $${IOU}_{3}=\frac{{A}_{1}\cap {A}_{2}\cap {A}_{3}}{{A}_{1}\cup {A}_{2}\cup {A}_{3}}$$

2 $${IoU}_{2}=\frac{({A}_{1}\cap {A}_{2})\cup ({A}_{1}\cap {A}_{3})\cup ({A}_{2}\cap {A}_{3})}{{A}_{1}\cup {A}_{2}\cup {A}_{3}}$$

3 $${IoU}_{1}=1- {(IoU}_{2}+{IoU}_{3})$$

### Simulation of segmentation variability

In addition to manual image segmentation, we expanded our approach by integrating Monte Carlo dropout (MCD) into an in-house U-Net model to simulate segmentation variability. The model, initially trained for short-axis T1 map LV myocardium segmentation, was subjected to inference with dropout probabilities (dp) ranging from 0 to 0.3, incremented by 0.02 to reflect real-world segmentation variability (IoU_1_ + IoU_2_) > 0.7 [21]. MCD leverages model uncertainty to produce valid segmentation masks with small variations that scale according to the dropout. This approach provides a straightforward way to repurpose a previously trained segmentation model to generate valid segmentation masks with varying degrees of segmentation variability, enabling us to study how these variations affect RTF repeatability. For each dp and T1 map, three segmentation masks (A1, A2, and A3) were generated, following the same procedure as in manual segmentation. The IoU values for each set of masks were averaged across the dataset for each dp and the manual segmentations. RTFs were then extracted from each mask and analyzed as described in the following sections.

### Radiomic feature extraction

We employed PyRadiomics [1, 7], an open-source Python package for the extraction of radiomic features from medical images. We extracted eleven sets of 93 features from each image. These 93 features can be conveniently organized into six feature families: the first-order features and five second-order feature families that are computed from specialized matrices GLCM = gray-level co-occurrence matrix, GLDM = gray-level dependence matrix, GLRLM = gray-level run length matrix, GLSZM = gray-level size-zone matrix, NGTDM = neighboring gray-tone-difference matrix. The term “eleven” pertains to the preprocessing filtering operations before feature extraction. These filtering operations can be categorized into two main groups. The first group involves point processing-based filters, which process pixels individually, including Original, Square, Square root, Logarithm, and Exponential filters. The second group involves neighborhood operation-based filters, which manipulate pixel values based on neighboring pixels within a defined local region or “neighborhood.” These filters include Gradient LBP2D = local binary pattern 2D, wavelet-HH = wavelet high-pass filter applied in horizontal and vertical directions, wavelet-HL = wavelet high- and low-pass filters applied in horizontal and vertical directions, wavelet-LH = wavelet low- and high-pass filters applied in horizontal and vertical directions, and wavelet-LL = wavelet low-pass filter applied in horizontal and vertical directions. Most filters did not require any settings; hence, they were used as implemented in pyradiomics. We performed preliminary studies for the LBP2D and wavelet filters to identify the best settings. Online Resource 1, Tables S2 and S3 provide a description of the filter classes and feature class and their settings used in this study. All images were resampled to 1 mm by 1 mm in-plane resolution before feature extraction.

### Statistical analysis

The Intraclass Correlation Coefficient (ICC) was employed as a reliability index to quantify RTF robustness [22]. ICC measures the proportion of variance in a set of measurements attributed to the variance of interest.

4 $$reliability\ index (ICC)=\frac{variance\ of\ interest}{variance\ of\ interest\ +\ unwanted\ variance}$$

The computation of ICC was carried out using hierarchic modeling, employing the Pingouin statistical package version 0.5.3. in python. The models employed for assessing repeatability and reproducibility are elaborated in subsequent sections. Each RTF’s ICC value was categorized as excellent (ICC ≥ 0.95), good (0.95 > ICC ≥ 0.75), moderate (0.75 > ICC ≥ 0.5), or poor (ICC < 0.5). Subsequently, for both reproducibility and repeatability assessments, the proportion of features in each category is reported for each feature class and filter class.

#### Repeatability

We defined repeatability as the degree to which multiple measures of RTFs, acquired under identical conditions, agree with each other. To assess this, we used ICC(2,1) to evaluate the absolute agreement between RTFs extracted from three independent segmentation runs (run1, run2, and run3), where the segmentation process was the only varying factor between the different runs. Since both the study participants and the segmentation process can introduce variability, we selected a two-way random effects model. This model was chosen because it accounts for two sources of randomness: (1) the participants, treated as a random effect to generalize findings beyond the specific individuals in the study, and (2) the segmentation runs, considered random to capture the variability inherent in the segmentation process itself. By using this model, we can reliably estimate the repeatability of a single RTF measurement from one segmentation run (ICC(2,1)) while ensuring that the results are generalizable to a broader population of segmenters exhibiting similar variability. Our model choice also allows us to measure the repeatability of k-averaged measures through ICC(2,k) (see Online Resource 1 for these additional results). Separate ICC values were calculated for the 1.5 T and 3 T scans, with results reported individually for each scanner type.

#### Reproducibility

We defined reproducibility as the degree to which the same measure of RTF acquired by scanners at different field strengths (1.5 T vs 3 T) is consistent. To evaluate this consistency, we employed the ICC(3,1). Our analysis acquired paired RTFs from the same patient, with segmentation performed by a single expert. This setup presents two primary sources of variation: (1) the differences in RTF values attributable to field strength and (2) the variability arising from differences among study participants. We selected a two-way mixed effects model for comparing texture features extracted from images obtained at 1.5 T and 3 T for the following reasons. First, this model treats the participants as random effects, enabling our findings to be generalized beyond the specific individuals included in the study. The differences in magnetic field strength are considered fixed effects. This approach allows us to estimate the repeatability of a single RTF measurement derived from either the 1.5 T or 3 T scanner. Additionally, the model provides the capability to assess the reproducibility of k-averaged measures through the ICC(3,k) (see Online Resource 1 materials for detailed results).

## Results

**Fig. 2:**
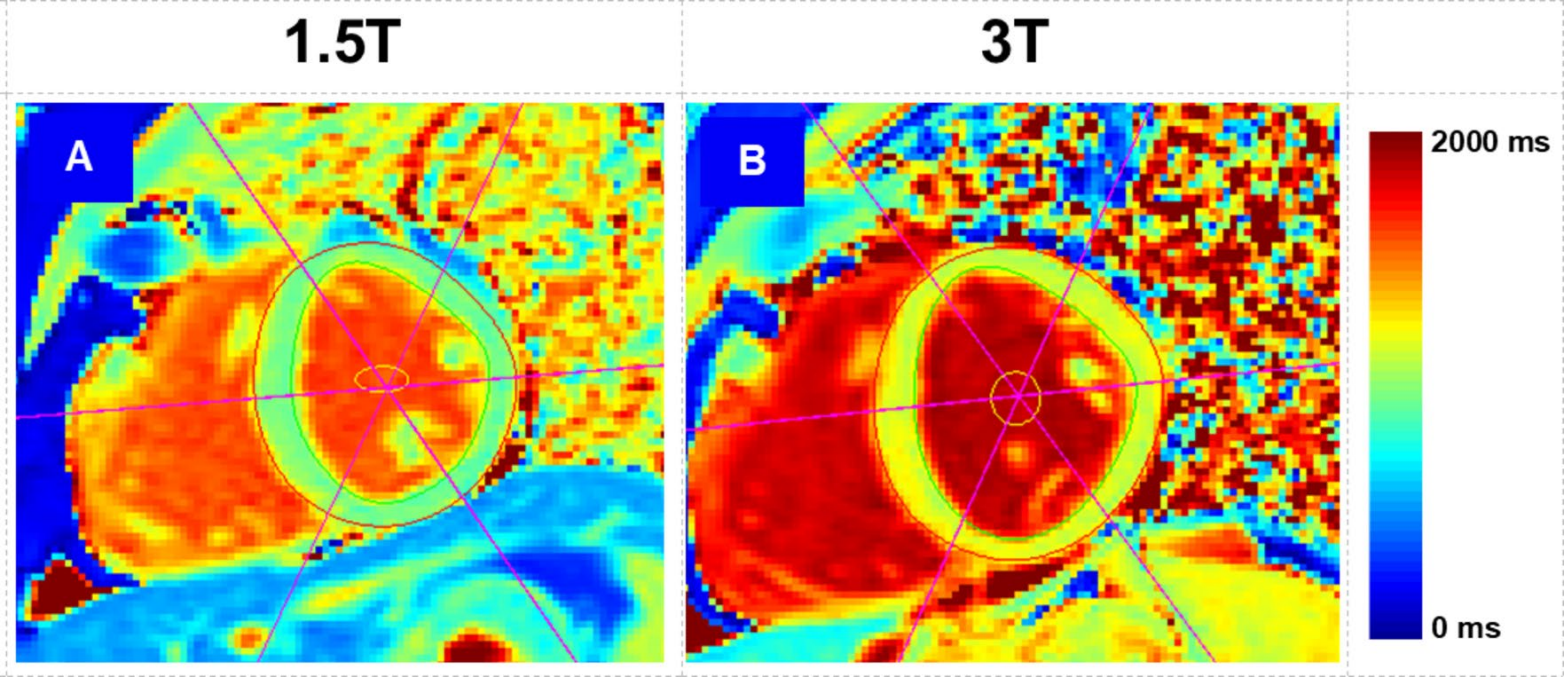
A sample paired T1 map acquired at different field strengths. Representative mid-short axis LV parametric maps acquired from a 33-year-old male participant and the contours drawn in one segmentation run. A is a 1.5 T T1 map, B is a 3 T T1 map. Apparent in the image is the difference in signal intensity levels. The 3 T map appears to have a higher signal intensity level, indicating that features such as mean T1 are not the same for both images

A total of 45 paired CMRs (1.5 T vs. 3 T) scans acquired from 15 healthy participants were utilized in this analysis. Figure 2 shows a representative pair of mid-cavity short-axis T1 maps.

### Similarity of segmented ROIs

**Fig. 3:**
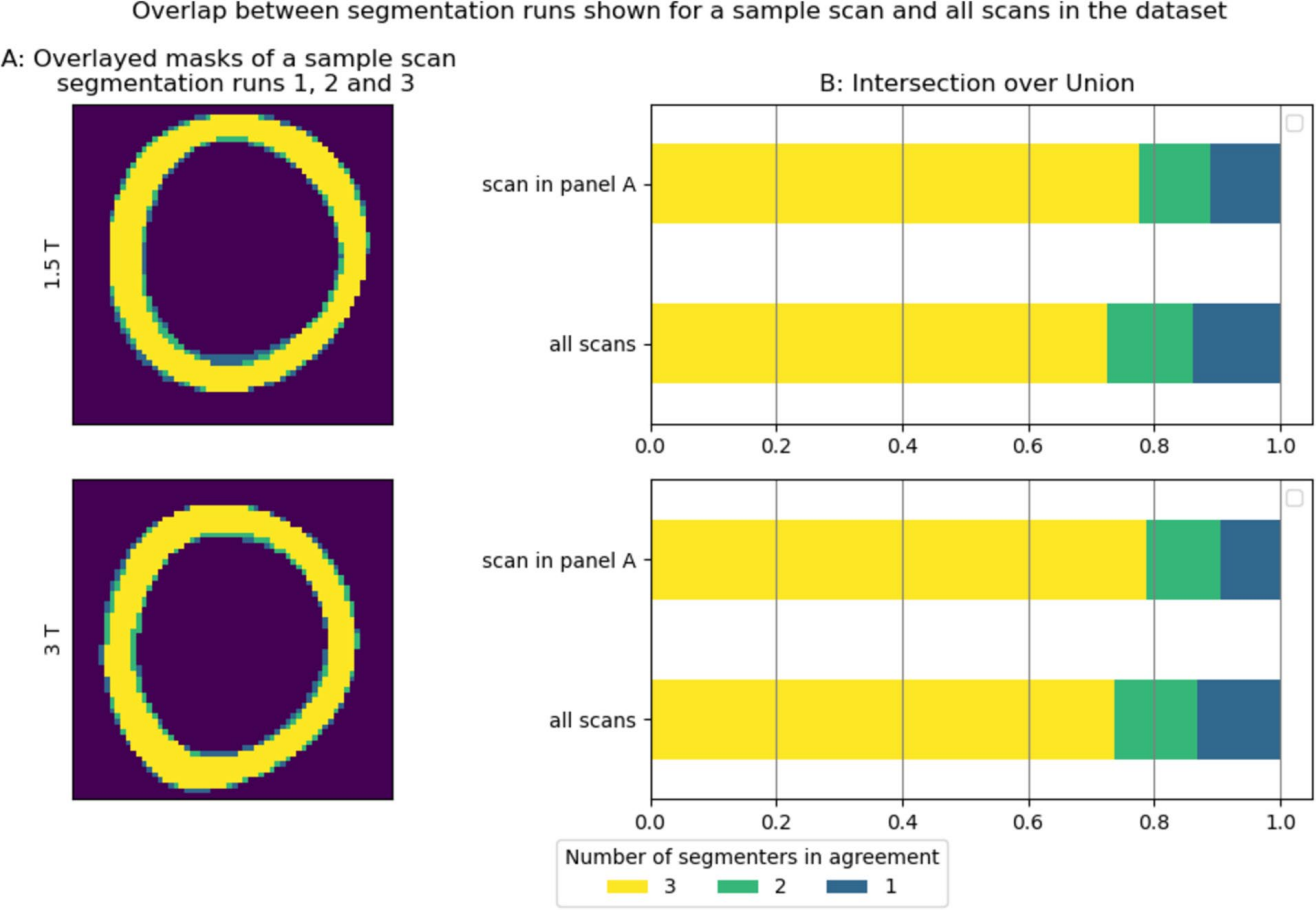
A figure showing the extent of agreement between the three segmentation runs. Panel A shows the overlap between the case example maps drawn by the three segmentation runs, color coding the areas that have achieved consensus between the three runs (3), two runs (2), and no agreement (1). Panel B shows the proportion of ROI that is agreed on by three, two, and only one segmentation run for the scan in Panel A, an average for the entire dataset

The agreement between manual segmentation runs is illustrated in Fig. 3. In 3A, the segmentation masks extracted from a selected scan at both 1.5 T and 3 T are displayed, showcasing the extent of overlap between all three segmentation runs (3), the additional area common to only two runs (2), and the region unique to each run (1) for each field strength. This visualization provides a clear depiction of the agreements among segmentation runs.

The IoU values for the masks in Fig. 3A and the average IoU values across the entire dataset for each field strength are shown in Fig. 3B. The IoU values for the scan in Fig. 3A are intended to assist our readers in interpreting the average values across the dataset.

For the 1.5 T scans, the average IoU values across the entire dataset were as follows: 0.725, or 72.5%, of the pixels captured by all segmentation runs, were common to run1, run2, and run3, indicating a substantial agreement (Fig. 3B, all scans, 1.5 T). Additionally, 13.7% of the pixels captured in all segmentation runs were agreed upon by two segmentation runs, while 13.8% did not reach consensus.

A similar level of consensus was observed among the 3 T masks. An average IoU value of 0.738 or 73.8% was achieved, signifying a robust agreement across all segmentation runs. Moreover, 13.1% of the segmented pixels were agreed on by two segmentation runs, while 13.1% showed disagreement among all segmentation runs. There were no observed differences in IoU_3_ values across 1.5 T and 3 T scans (0.725 vs 0.738).

### Reproducibility across scanners (1.5 T vs 3 T)

A total of 1023 RTFs were initially extracted and evaluated for reproducibility across the 1.5 T and 3 T scanners. However, “lbp-2D_firstorder_Maximum,” was identified as numerically unstable, consistently yielding the same value across all scans and field strengths. Consequently, this feature was excluded from further analysis, resulting in 1022 RTFs for subsequent investigation.

No RTFs demonstrated excellent reproducibility, defined by ICC values exceeding 0.95. The analysis revealed that 76 RTFs (7.44% of the 1022 RTFs) displayed good reproducibility. Additionally, 402 RTFs (39.33%) exhibited moderate reproducibility, while 544 features (53.23%) demonstrated poor reproducibility (Table 1). Notably, the feature with the highest reproducibility was identified as “gldm_GrayLevelNonUniformity” with an ICC of 0.879 (95% CI = 0.79–0.93).

**Table 1:** A table showing the proportion of RTFs that fell in each category of reproducibility across the 1.5 T and 3 T scanners and the repeatability across segmentation runs for 1.5 T and 3 T

Categories	Reproducibility		Repeatability (manual segmentation)			
	1.5 T Vs 3 T		1.5 T		3 T	
	*Num of RTFs*	*%*	*Num of RTFs*	*%*	*Num of RTFs*	*%*
Excellent	0	0.00	334	32.68	322	31.51
Good	76	7.44	414	40.51	553	54.11
Moderate	402	39.33	176	17.22	131	12.82
Poor	544	53.23	98	9.59	16	1.56

**Fig. 4:**
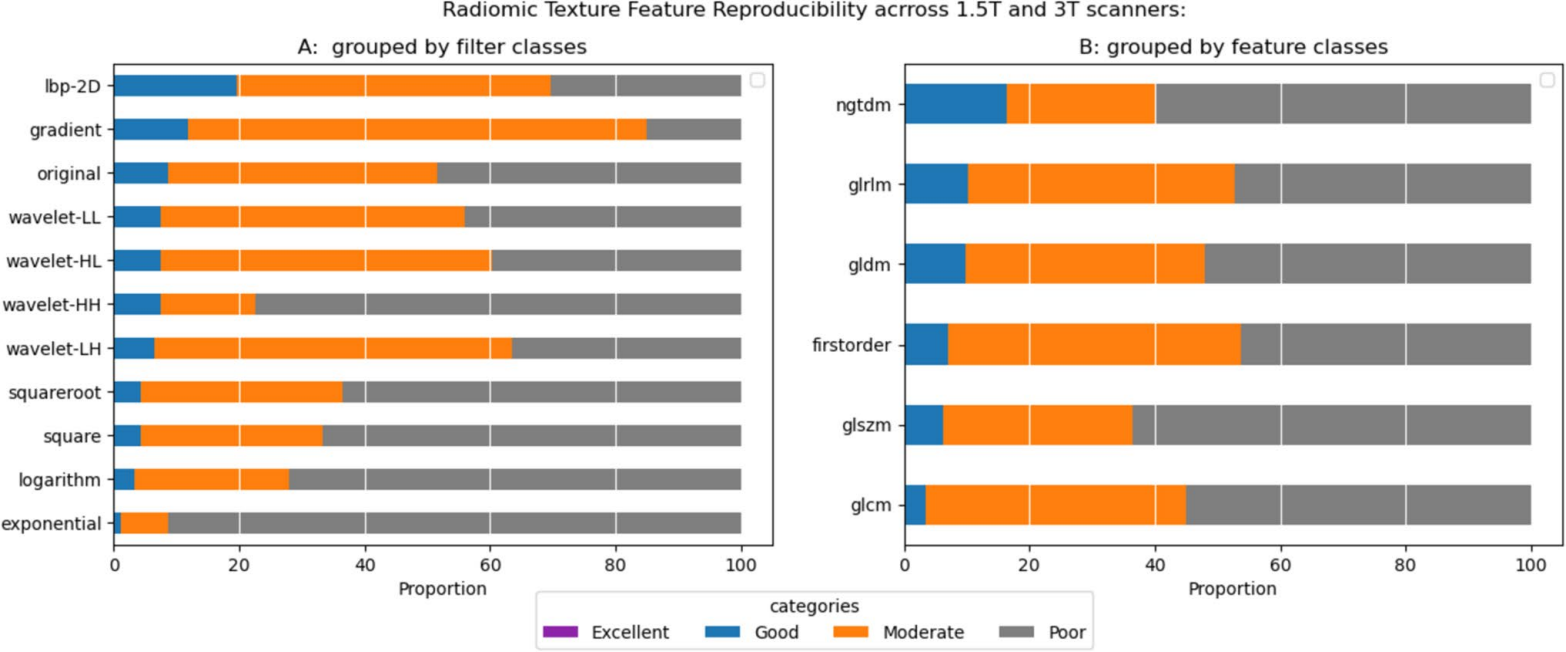
Reproducibility of RTFs. A display of the proportion of RTFs from **A** each filter class, **B** each feature class that fall in each category of reproducibility

Filter Class Level: Fig. 4A shows the reproducibility at the filter class level, ranked from the highest to the lowest by the percentage of features with good reproducibility. No filter class produced an RTF with excellent reproducibility across 1.5 T and 3 T scanners. The percentage of RTFs with good reproducibility varied across filter classes, ranging from approximately 1% (exponential, n = 1) to 20% (lbp-2D, n = 63). Features with moderate reproducibility ranged from 8% (exponential, n = 7) to 73% (gradient, n = 68), while those with poor reproducibility ranged from 12% (gradient, n = 14) to 91% (exponential, n = 85). Table 2 highlights the most reproducible features in each filter class. The glrlm_GrayLevelNonUniformity exhibited dominance in reproducibility, being the most reproducible feature in 6 filter classes (original, squareroot,wavelet-HL and LH, logarithm, and square). The gldm_DependenceNonUniformity and gldm_GrayLevelNonUniformity were the most reproducible features in 2 filter classes each: (lbp-2D, exponential) and (gradient,wavelet-LL). The glrlm_RunLengthNonUniformity was the most reproducible feature in the wavelet-HH filter class.

**Table 2:** A table showing the most reproducible RTF in each filter class

Filter name	Feature name	ICC(3,1)	95% CI
Gradient	gldm_GrayLevelNonUniformity	0.879	[0.790 0.930]
Original	glrlm_GrayLevelNonUniformity	0.878	[0.790 0.930]
Squareroot	glrlm_GrayLevelNonUniformity	0.878	[0.790 0.930]
Wavelet-LL	gldm_GrayLevelNonUniformity	0.874	[0.780 0.930]
Wavelet-HL	glrlm_GrayLevelNonUniformity	0.873	[0.780 0.930]
lbp-2D	gldm_DependenceNonUniformity	0.866	[0.770 0.920]
Wavelet-LH	glrlm_GrayLevelNonUniformity	0.857	[0.750 0.920]
Logarithm	glrlm_GrayLevelNonUniformity	0.854	[0.750 0.920]
Square	glrlm_GrayLevelNonUniformity	0.840	[0.730 0.910]
Wavelet-HH	glrlm_RunLengthNonUniformity	0.836	[0.720 0.910]
Exponential	gldm_DependenceNonUniformity	0.769	[0.620 0.870]

Figure 4B further breaks down the results at the feature class level. The percentage of RTFs with good reproducibility ranged from 3.4% (glcm) to 16% (glrlm). Similarly, the percentage of features with moderate reproducibility ranged from 23% (ngtdm) to 46% (firstorder), while features with poor reproducibility ranged from 46% (firstorder) to 63% (glszm).

### Repeatability of RTFs across segmentation runs

**Fig. 5:**
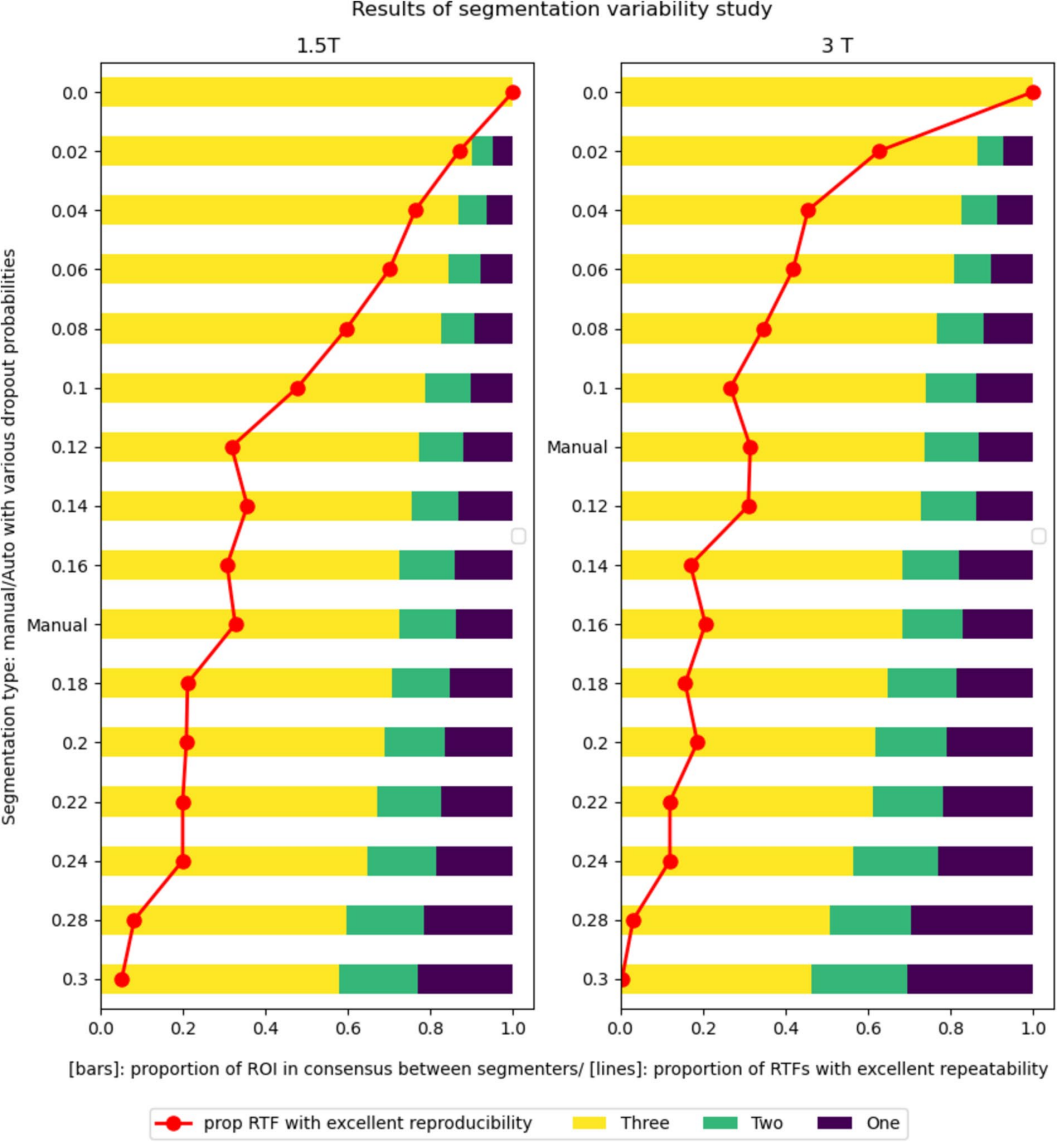
A plot of the results showing the sensitivity of RTFs to variations in image segmentation. The bars show the proportion of the segmented ROI that attained consensus between three, two and only one segmentation runs. The lines show the corresponding number of RTFs that fell in each category of repeatability

Figure 5 shows the results for the effects of segmentation variability on the repeatability of RTFs, illustrating a discernible trend. As the dropout probability (dp) increases, there is a corresponding decrease in the proportion of ROI pixels achieving consensus across the three segmentation runs (IoU_3_). This, in turn, results in a decline in RTFs with excellent repeatability for both 1.5 T and 3 T scans. The graph shows that with dp = 0, all three masks are perfectly Identical (IoU3 = 1.0), resulting in 100% or (proportion = 1.00) of all RTFs being repeatable. This proportion gradually decreases in both 1.5 T and 3 T images. At dp = 0.3, where IoU3 approaches 0.5, only about 5% or (proportion = 0.05) of RTFs produce excellent repeatability.

Table 2 (Repeatability) compares RTFs extracted from three manual segmentation runs (run1, run2, and run3) for repeatability. From the 1.5 T scans, the proportion of features, 32.68% (n = 334), exhibited excellent repeatability. Furthermore, 40.51% (n = 414) displayed good repeatability, 17.22% (n = 176) and 9.58% (n = 31) demonstrated moderate and poor repeatability, respectively. The most repeatable feature was gradient_gldm_GrayLevelNonUniformity, with an ICC of 0.993 (95% CI 0.990–1.000).

For the 3 T RTFs, we observed a similar yet slightly improved repeatability profile. A total of 31.56% (n = 322) of features displayed excellent repeatability, showcasing high consistency in the 3 T scans. Additionally, 54.11% (n = 553) exhibited good repeatability, while 12.81% (n = 131) and 1.56% (n = 16) showed moderate and poor repeatability, respectively. The most repeatable RTF from the 3 T scans was gradient_gldm_LargeDependenceLowGrayLevelEmphasis, with an ICC of 0.995 (95% CI 0.990–1.000). The repeatability patterns were further analyzed for each field strength at the filter class and feature class level.

#### Repeatability of 1.5 T features

**Fig. 6:**
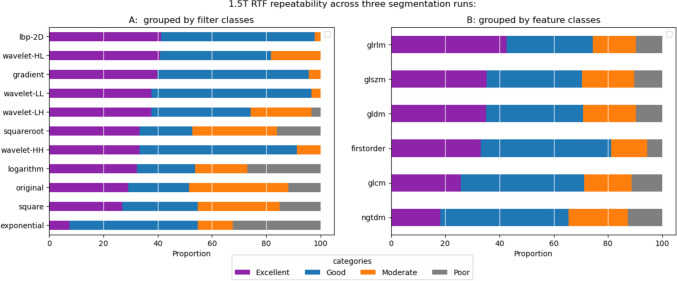
Repeatability of RTFs. A chart showing the proportion of 1.5 T RTFs from each **A** filter class and **B** feature class that fall in each category of repeatability

Breaking down the repeatability of RTFs from 1.5 T scans across all segmentation runs, we observed varying degrees of repeatability with excellent repeatability at the feature class level, ranging from approximately 8% (exponential, n = 7) to 41% (lbp-2D/wavelet-HL, n = 38). The proportion of features with good repeatability ranged from 19% (squareroot, n = 18) to 59% (wavelet-LL, n = 55), while the percentage of RTFs with moderate or worse repeatability varied from 2% (lbp-2D, n = 2) to around 48% (original, n = 45). At the feature class level, the range of features with excellent repeatability extended from 18% (ngtdm) to 42% (glrlm), while features with good repeatability ranged from 31% (grlm) to 48% (ngtdm) (Fig. 6). The most repeatable feature in each filter category is detailed in Table 3. The “firstorder_Median” emerged as the most repeatable feature in most filter classes, including squareroot, original, and wavelet-LL.

**Table 3:** A table showing the most Repeatable RTF in each filter class for 1.5 T and 3 T scans

Filter name	Feature name	ICC(2,1)	95% CI
1.5 T			
Gradient	gldm_LargeDependenceLowGrayLevelEmphasis	0.994	[0.990 1.000]
Squareroot	firstorder_Median	0.991	[0.980 0.990]
Wavelet-LL	firstorder_Median	0.990	[0.980 0.990]
Original	firstorder_Median	0.990	[0.980 0.990]
Logarithm	firstorder_Mean	0.990	[0.980 0.990]
Square	gldm_GrayLevelNonUniformity	0.989	[0.980 0.990]
lbp-2D	glszm_SizeZoneNonUniformity	0.988	[0.980 0.990]
Wavelet-LH	gldm_GrayLevelNonUniformity	0.982	[0.970 0.990]
Wavelet-HL	glrlm_RunVariance	0.981	[0.970 0.990]
Wavelet-HH	glcm_Idm	0.979	[0.970 0.990]
Exponential	firstorder_Median	0.967	[0.950 0.980]
3 T			
Gradient	gldm_GrayLevelNonUniformity	0.995	[0.990 1.000]
Exponential	glcm_ClusterProminence	0.994	[0.990 1.000]
Original	firstorder_Median	0.992	[0.990 1.000]
Wavelet-LH	gldm_GrayLevelNonUniformity	0.992	[0.990 1.000]
Square	gldm_GrayLevelNonUniformity	0.992	[0.990 1.000]
Wavelet-HH	gldm_GrayLevelNonUniformity	0.991	[0.980 0.990]
Wavelet-HL	gldm_GrayLevelNonUniformity	0.990	[0.980 0.990]
Logarithm	gldm_GrayLevelNonUniformity	0.990	[0.980 0.990]
Squareroot	gldm_GrayLevelNonUniformity	0.990	[0.980 0.990]
Wavelet-LL	gldm_GrayLevelNonUniformity	0.990	[0.980 0.990]
lbp-2D	glszm_SizeZoneNonUniformity	0.987	[0.980 0.990]

#### Repeatability of 3 T features

**Fig. 7:**
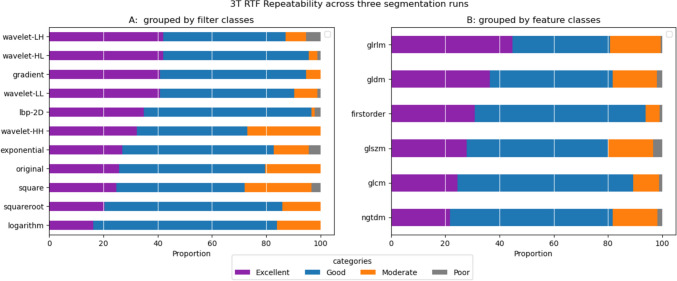
Repeatability of RTFs. A chart showing the proportion of 3 T RTFs from each **A** filter class and **B** feature class that fall in each category of repeatability

The percentage of 3 T RTFs with excellent repeatability ranged from about 16% (logarithm, n = 15) to 42% (wavelet-HL /wavelet-LH, n = 39). Features with good repeatability ranged from 40% (wavelet-HH, n = 38) to 68% (logarithm, n = 63), while RTFs with moderate or worse repeatability fell between 3% and about 28% (square, n = 26). At the feature class level, the features with excellent repeatability extended from 21% (ngtdm) to 45% (glrlm). Features with good repeatability were observed at percentages ranging from 35% (grlm) to 65% (glcm) (Fig. 7). Notably, “gldm_GrayLevelNonUniformity” emerged as the most repeatable feature in most filter classes, including squareroot, square, gradient, logarithm, and all wavelet filters.

## Discussion

Radiomic texture features have emerged as a promising frontier in the development of CMR imaging biomarkers. As we venture into this realm, a thorough understanding of the effects of variations in the radiomic workflow becomes very important in assessing the reliability of RTFs across multiple imaging settings and establishing baseline values. However, the consistency and reproducibility of these RTFs across different imaging scenarios, particularly at varying field strengths, remains unexplored. To address this critical concern, we performed this retrospective study to assess the reproducibility of RTFs across scanners with different field strengths (1.5 T vs 3 T) run at their standard clinical protocol settings and the effects of segmentation variability on the repeatability of these features. We analyzed 45 paired T1 LV myocardial scans at each field strength acquired from 15 healthy participants for reproducibility and repeatability.

Utilizing the IoU3 metric to assess concordance among segmentation runs, our findings reveal a decrease in the proportion of myocardial RTFs with excellent repeatability as IoU3 decreases. When we broke down our results for the manual segmentation at the filter class and feature class levels, we observed consistent levels of agreement across segmentation runs for both 1.5 T and 3 T scans. Notably, 86–87% of pixels encompassed by the three manual segmentation runs achieved a consensus among at least two segmentation runs (IoU_3_ + IoU_2_). With the above agreement in image segmentation, more than 73% of RTFs extracted from each filter had good or excellent repeatability in both 1.5 T and 3 T images, indicating that a large majority of RTFs are robust to small levels of variability in image segmentation. The lbp-2D filter produced the highest number of features with good or better repeatability across segmentation runs for both 1.5 and 3 T images, while the first-order features were the most repeatable. While the observation of RTFs with excellent reproducibility across different field strengths was not evident, we did find that over 7% of RTFs demonstrated good reproducibility while almost 40% had moderate reproducibility. Notably, we observed a dominance of neighborhood filters when considering the most robust filter classes, producing the most reproducible features across field strengths. Specifically, gradient, LBP-2D, wavelet-LH, wavelet-LL, and wavelet-HL were the top five in terms of reproducibility. Conversely, point operation-based filters such as square root, square, logarithm, and exponential were predominant among the filters with relatively lower reproducibility. The Gray Level Nonuniformity feature emerged as the most reproducible feature across various filter categories. These results have important implications for both the theoretical and practical aspects of radiomics analysis.

From our repeatability analysis, a substantial number of RTFs have demonstrated notable levels of robustness when subjected to minor variations in image segmentation. While this initial finding may be considered encouraging, it is crucial to underscore the potential fragility of repeatability. Even slight increases in the proportion of ROI pixels that do not reach a consensus between different segmenters can result in significant reductions in repeatability, a trend supported by prior studies [7, 14]. This observation highlights the sensitivity of repeatability outcomes to segmentation variability. To overcome this limitation, highly accurate and specific automated CMR myocardial image segmentations could be employed in the future to reduce inter-observer and intra-observer variability and improve the accuracy and repeatability of radiomic texture features.

Theoretical considerations suggest that certain features are less susceptible to variations in image parameters and more reflective of the intrinsic tissue properties they represent [23]. Our findings in the reproducibility analysis strongly align with these foundational principles. Notably, the radiomic texture features derived from neighborhood operation filters, including Wavelet LL, LH, HL, gradient, and LBP2D, were more reproducible across field strength. These filters are particularly notable for their capacity to account for intensity changes in the pixel neighborhood, allowing them to capture relative changes in pixel intensity rather than absolute values. This unique characteristic renders these features notably robust when confronted with fluctuations in imaging parameters, such as field strength. The implications of this robustness extend to their applicability in both research and clinical settings, where consistent feature performance is paramount.

The findings of our study will help to further our understanding of the effects of variations in the radiologic pipeline on the reproducibility and repeatability of radiomic texture features. Structural features such as gray-level nonuniformity and neighborhood filters have been identified to be more resistant to changes in the pipeline. The identification of robust RTFs holds significant promise for advancing the development of clinical biomarkers and enhancing machine-learning models in cardiovascular imaging. By establishing reliable and reproducible RTFs, we can improve the predictive accuracy of machine learning algorithms, enabling them to better stratify patients based on their risk profiles and clinical outcomes. These robust features facilitate the validation of clinical biomarkers by providing objective measures that correlate consistently with disease characteristics, thereby supporting their integration into clinical practice. Additionally, incorporating robust RTFs can minimize variability, ensuring that the diagnostic conclusions drawn from machine learning models are accurate and clinically relevant. The establishment of robust RTFs paves the way for personalized medicine approaches, where treatment decisions are informed by a deeper understanding of individual patient profiles and disease mechanisms, leading to improved patient care and outcomes.

There are a number of limitations that should be considered when interpreting the presented findings. This study was a single-center, single-vendor retrospective analysis performed on a small cohort of healthy participants. We did not present any subgroup analysis on how the identified RTFs will perform in the presence of disease conditions. The images used in the study were acquired from 1.5 T and 3 T scanners run at their institutional clinical protocol settings. While it would be desirable to fix all imaging parameters and vary only field strength, this is not practical in this case as some imaging parameters, such as repetition time, were selected to be commensurate with the patient’s heart rate. Furthermore, the 1.5 T Siemens Area and 3 T Vida scanners used in this study are not considered benchmark systems for their respective field strengths and were, therefore, treated as fixed effects in our analysis comparing reproducibility across field strengths. While it is practically challenging to evaluate patients across all scanner platforms or identify single scanners that are considered benchmarks for their field strengths, these specific models were chosen due to their availability at our institution and their common use in clinical practice. As such, the findings reflect real-world clinical scenarios. Despite these limitations, we believe our findings provide valuable insights into the filters and feature classes that better capture intrinsic tissue characteristics under varying image acquisition parameters.

## Conclusions

In conclusion, our study unveils significant variations in myocardial RTFs acquired from the same patient using clinical scanners with different field strengths in normative settings. While no RTF achieved excellent reproducibility, 7% demonstrated good reproducibility, while about 40% had moderate reproducibility. The investigation underscores RTFs’ sensitivity to minor segmentation variations. Manual segmentation produced more than 73% of RTFs with good or better repeatability and promises of improvement with more consistent segmentation. Neighborhood operation-based filters and structural RTFs emerged as the more robust myocardial RTFs. These findings carry implications for texture feature-based markers and machine learning pipelines, emphasizing the crucial role of selecting robust features for enhanced reliability in medical imaging analyses.

## Supplementary Information

Below is the link to the electronic supplementary material.

Supplementary file1 (PDF 2403 KB)

Supplementary file2 (PDF 89 KB)

Supplementary file3 (PDF 88 KB)

## Data Availability

The data and segmentation model that support the findings of this study are available from the corresponding author, JHJ, upon reasonable request.

## Abbreviations

CCC: Concordance correlation coefficient
CMR: Cardiovascular magnetic resonance
IoU: Intersection over union
GLCM: Gray level co-occurrence matrix
GLDM: Gray level dependence matrix
GLRLM: Gray level run length matrix
GLSZM: Gray level size zone matrix
ICC: Intraclass correlation coefficient
IRB: Institutional review board
LBP: Local binary pattern
MOLLI: Modified look-locker inversion recovery
NGTDM: Neighboring gray tone difference matrix
ROI: Region of interest
RTF: Radiomic texture feature
SCMR: Society of cardiovascular magnetic resonance imaging
