# Supplementary material for: Effect of magnetic field strength and segmentation variability on the reproducibility and repeatability of radiomic texture features in cardiovascular magnetic resonance parametric mapping
Source: Int J Cardiovasc Imaging. 2025 Jan 8;41(2):325–37. doi: 10.1007/s10554-024-03312-7 (PMC11811471; doi:10.1007/s10554-024-03312-7)
Supplement: Supplementary file 1 — Supplementary file1 (PDF 2403 KB) [file 10554_2024_3312_MOESM1_ESM.pdf]

Supplementary material

for

Effect of magnetic field strength and segmentation variability on the reproducibility and repeatability of radiomic texture features in cardiovascular magnetic resonance parametric mapping

Pascal Yamlome, MS[a] and Jennifer H. Jordan, PhD[a,b]

From the [a]Department of Biomedical Engineering, College of Engineering and [b] Division of Cardiology, Pauley Heart Center at Virginia Commonwealth University, Richmond, Virginia, USA

Technical Image Acquisition Parameters

Table S1. A table showing the detailed image acquisition parameters used for scanning each participant in the study

Patient ID	Field Strength	TE	TR	Acquisition Matrix	image size	pixel Spacing/ Inplane Res	FOV	Percent Phase FOV
1	1.5	1.00	314.20	256, 144	218, 256	1.40625, 1.40625	306, 360	85.16
2	1.5	1.00	314.20	256, 144	218, 256	1.40625, 1.40625	306, 360	85.16
3	1.5	1.00	314.20	256, 144	256, 218	1.40625, 1.40625	306, 360	85.16
4	1.5	1.00	314.20	256, 144	256, 218	1.40625, 1.40625	306, 360	85.16
5	1.5	1.00	314.20	256, 144	256, 218	1.40625, 1.40625	306, 360	85.16
6	1.5	1.00	314.20	256, 144	256, 218	1.40625, 1.40625	306, 360	85.16
7	1.5	1.00	314.20	256, 144	256, 218	1.40625, 1.40625	306, 360	85.16
8	1.5	1.00	314.20	256, 144	256, 218	1.40625, 1.40625	306, 360	85.16
9	1.5	1.00	314.20	256, 144	256, 218	1.40625, 1.40625	306, 360	85.16
10	1.5	1.00	314.20	256, 144	256, 218	1.40625, 1.40625	306, 360	85.16
11	1.5	1.00	314.20	256, 144	256, 218	1.40625, 1.40625	306, 360	85.16
12	1.5	1.00	314.20	256, 144	256, 218	1.40625, 1.40625	306, 360	85.16
13	1.5	1.00	314.20	256, 144	218, 256	1.40625, 1.40625	306, 360	85.16
14	1.5	1.00	314.20	256, 144	256, 218	1.40625, 1.40625	306, 360	85.16
15	1.5	1.00	314.20	256, 144	218, 256	1.40625, 1.40625	306, 360	85.16
1	3	1.00	287.60	192, 131	192, 162	1.87500, 1.87500	306, 360	84.38
2	3	1.10	319.30	256, 146	256, 220	1.40625, 1.40625	306, 360	85.94
3	3	1.10	311.20	256, 142	256, 214	1.40625, 1.40625	300, 360	83.59
4	3	1.10	311.20	256, 142	256, 214	1.40625, 1.40625	300, 360	83.59
5	3	1.10	311.20	256, 142	256, 214	1.40625, 1.40625	300, 360	83.59
6	3	1.00	283.10	131, 142	192, 162	1.87500, 1.87500	303, 360	84.38
7	3	1.10	316.70	256, 143	256, 216	1.40625, 1.40625	303, 360	84.38
8	3	1.10	311.20	256, 142	256, 214	1.40625, 1.40625	300, 360	83.59
9	3	1.10	321.60	256, 143	256, 216	1.32812, 1.32812	286, 340	84.38
10	3	1.10	317.70	256, 143	256, 216	1.36719, 1.36719	295, 350	84.38
11	3	1.10	311.20	256, 142	256, 214	1.40625, 1.40625	300, 360	83.59
12	3	1.10	311.20	256, 142	214, 256	1.40625, 1.40625	300, 360	83.59
13	3	1.10	322.00	256, 143	256, 216	1.28906, 1.28906	278, 360	84.38
14	3	1.10	311.20	256, 142	256, 214	1.40625, 1.40625	300, 360	83.59
15	3	1.10	311.20	256, 142	256, 214	1.40625, 1.40625	300, 360	83.59

Radiomic Texture Filter Classes

Table S2: A table of the Filters used to preprocess the input image before feature extraction, a brief description of how they transform the image, and the settings used, if any.

Filter name	Description	Settings
Point operations		
Original	This is the original image.	NA/default
Square	The square filter takes the square of image intensities and linearly scales them back to the original range, negating pixels that had a negative value in the original image. This filter enhances the image by emphasizing the high-intensity regions and suppressing the low-intensity regions.	NA/default
Square root	The square root filter takes the square root of the image intensities and linearly scales them back to the original range. This enhances the image by emphasizing the low-intensity regions and suppressing the high-intensity regions.	NA/default
Logarithm	The Logarithm filter takes the logarithm of the image intensities and linearly scales them back to the original range. This filter enhances the image by expanding the dynamic range of the low-intensity regions and compressing the dynamic range of the high-intensity regions.	NA/default
Exponential	The exponential filter takes the exponential of the image intensities and linearly scales them back to the original range. This filter enhances the image by expanding the dynamic range of the high-intensity regions and compressing the dynamic range of low-intensity regions.	NA/default
Neighborhood Operations		
Gradient	The Gradient filter calculates the gradient magnitude in the image, which represents the rate of change of the image intensity at each pixel. The Gradient filter enhances the image by highlighting the edges and boundaries of the regions of interest. This can help in feature extraction by identifying the texture and shape of the regions of interest.	NA/default
LBP2D	LBP stands for Local Binary Pattern, which is a texture descriptor used in computer vision. The LBP2D filter works by comparing the intensity of each pixel in the image with the intensity of its neighbors. If the intensity of the neighbor is greater than or equal to the intensity of the center pixel, a binary value of 1 is assigned to that neighbor. Otherwise, a binary value of 0 is assigned. The binary values are then concatenated to form a binary pattern, which is used to describe the texture of the region of interest. The LBP2d filter enhances the image by describing the local texture of the regions of interest.	Radius = 3<br>Num of samples = 12<br>Method = default
Wavelet HH, HL, LH, LL	The wavelet filter decomposes the input image into multiple frequency bands, each representing a different level of detail. This filter uses a selected wavelet to decompose the input image through the discrete wavelet transform (DWT) and uses the coefficients to extract features. We	Wavelet = rbio3.1

	decomposed each image into the LL, HH, HL, and LH subbands. The LL subband is a component of the wavelet transform that represents the low-frequency sub-band in the horizontal, vertical, and diagonal directions. The HH subband contains the finest information and is used to capture the diagonal details of the image. The HH subband is significant in wavelet decomposition because it contains the high-frequency components of the image, which are responsible for capturing the fine details of the image. The HL subband represents the high-frequency sub-band in the vertical direction, while the LH subband represents the high-frequency sub-band in the horizontal direction.	

Radiomic Texture Feature Families

Table S3: A table of the Radfiomic Texture Feature (RTF) families, a brief description of each feature family, and the number of RTFs in the family

Feature family	Description	Number of features
First-order	First-order features describe the distribution of voxel intensities within the image. These features are based on the image intensities themselves and are commonly referred to as intensity features.	19
GLCM	The Gray Level Co-occurrence Matrix (GLCM) records how often pairs of pixels with specific intensity values and spatial relationships occur within a neighborhood and in a given direction in the image	24
GLRLM	The Gray Level Run Length Matrix (GLRLM) is a 2D matrix where the rows represent different gray levels, and the columns represent different run lengths. A run is a sequence of consecutive pixels with the same gray-level value in a specific direction (e.g., horizontal, vertical, diagonal).	16
GLSZM	The Gray level size zone matrix (GLSZM) is a matrix that quantifies the distribution of connected regions of each gray level in the image based on their sizes. The rows represent different gray levels, and the columns represent different zone sizes. Each element (i, j) of the matrix represents the number of zones of size j for the gray level i in the image	16
NGTDM	The Neighboring gray-tone difference matrix (NGTDM) is based on the concept of how the gray levels of a pixel relate to the gray levels of its surrounding pixels. It quantifies the differences in gray levels between a central pixel and its neighboring pixels within a defined neighborhood	5
GLDM	The Gray level dependence matrix (GLDM) is a matrix that quantifies the occurrence of pairs of pixels with	14

	specific gray levels at a certain distance and direction. Each element (i, j) of the GLDM matrix represents the number of times a pixel with gray level i is adjacent to a pixel with gray level j at a certain distance and direction	

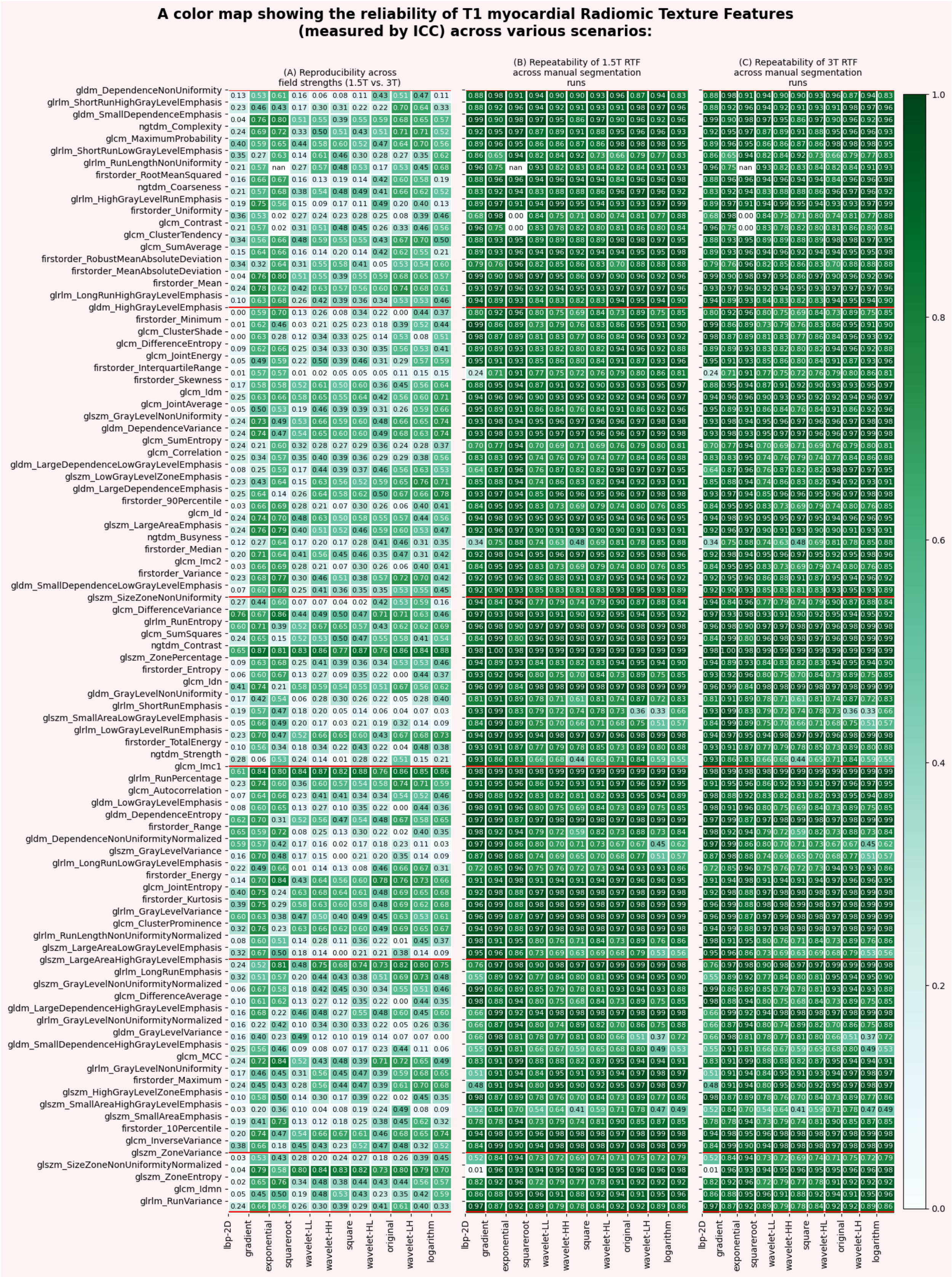

Figure S1. A color grid depicting the reliability of single measurements for each assessed myocardial radiomic texture feature, with pixels annotated by ICC values. (A) represents the reproducibility across field strengths (1.5T vs. 3T) measured by ICC(2,1). (B) illustrates the repeatability across manual segmentation runs for 1.5T scans, while (C) presents the repeatability for 3T scans, both measured by ICC(3,1).

Reliability of k-averaged Measures of RTFs

In practice, the reliability of any measured quantity can be enhanced by averaging multiple measurements, a concept grounded in the statistical law of large numbers. This principle suggests that as the number of measurements increases, the influence of random error decreases, yielding a more stable and accurate estimate. However, averaging may not always be practical, depending on the experimental setting. For example, in our reproducibility analysis, achieving higher reliability would require averaging radiomic texture feature (RTF) measurements across different magnetic field strengths (e.g., 1.5T and 3T), a method rarely applied in practice due to technical and clinical considerations. In contrast, in our repeatability study, reliability enhancement can be practically achieved by averaging RTF measurements acquired from multiple segmentation masks, allowing for improved stability of results within the same field strength. The subsequent figures illustrate the reliability of k-averaged measurements obtained in our experiments, highlighting the effects of averaging on the stability and consistency of RTF measurements.

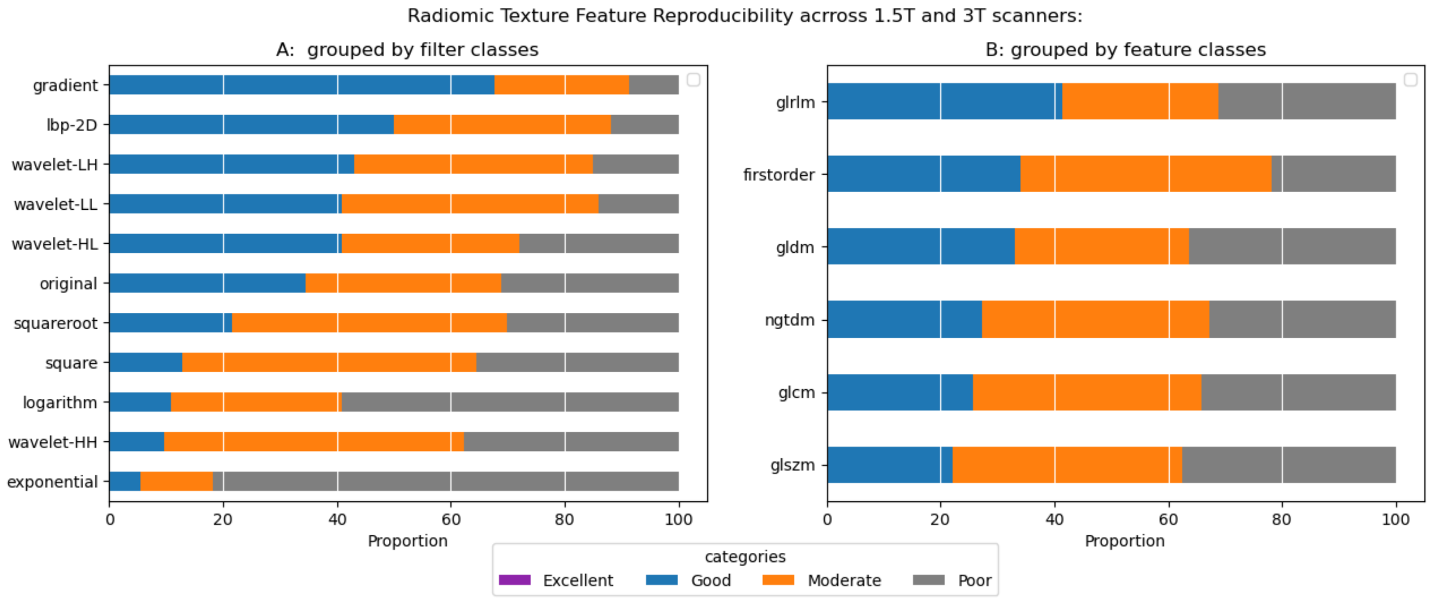

Figure S2 Reproducibility of k-averaged measures of RTF (1.5T vs 3T) assessed by ICC(2,k). A display of the proportion of RTFs from (A) each filter class, (B) each feature class that fall in each category of reproducibility

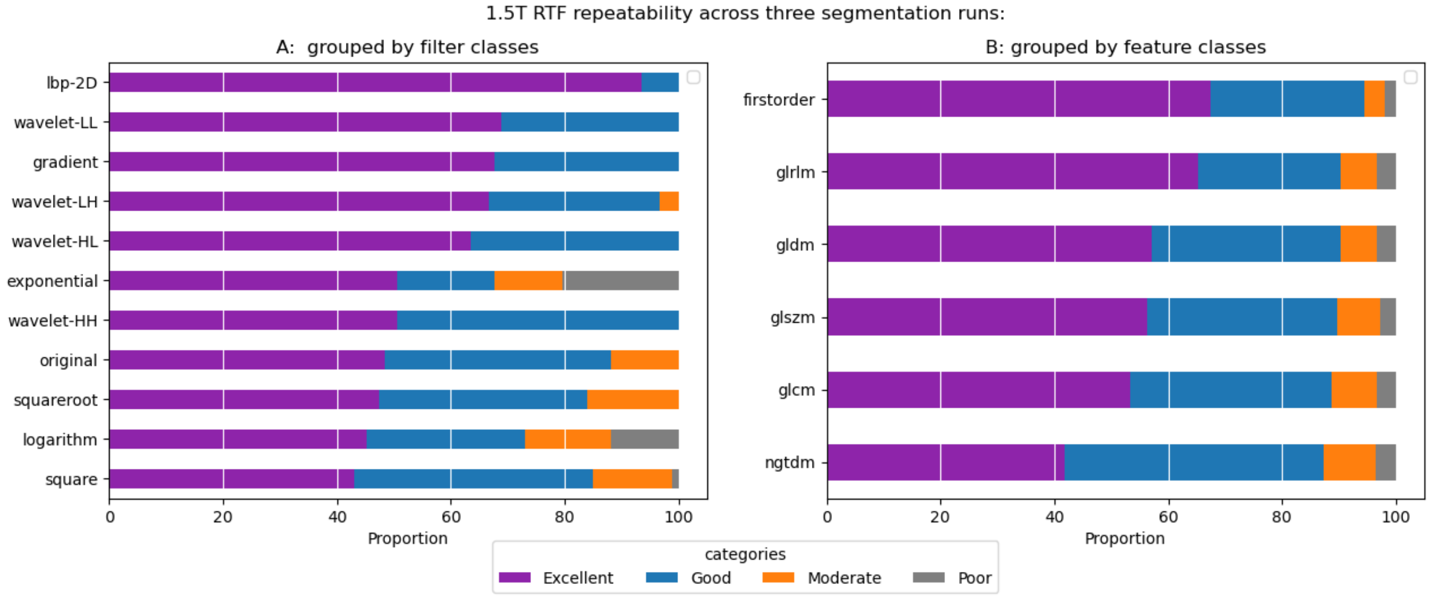

Figure S3 Repeatability of k-averaged measures of RTFs (segmentation mask from run1, run2, and run3) assessed by ICC(3,k). A chart showing the proportion of 1.5T RTFs from each (A) filter class and (B) feature class that fall in each category of repeatability

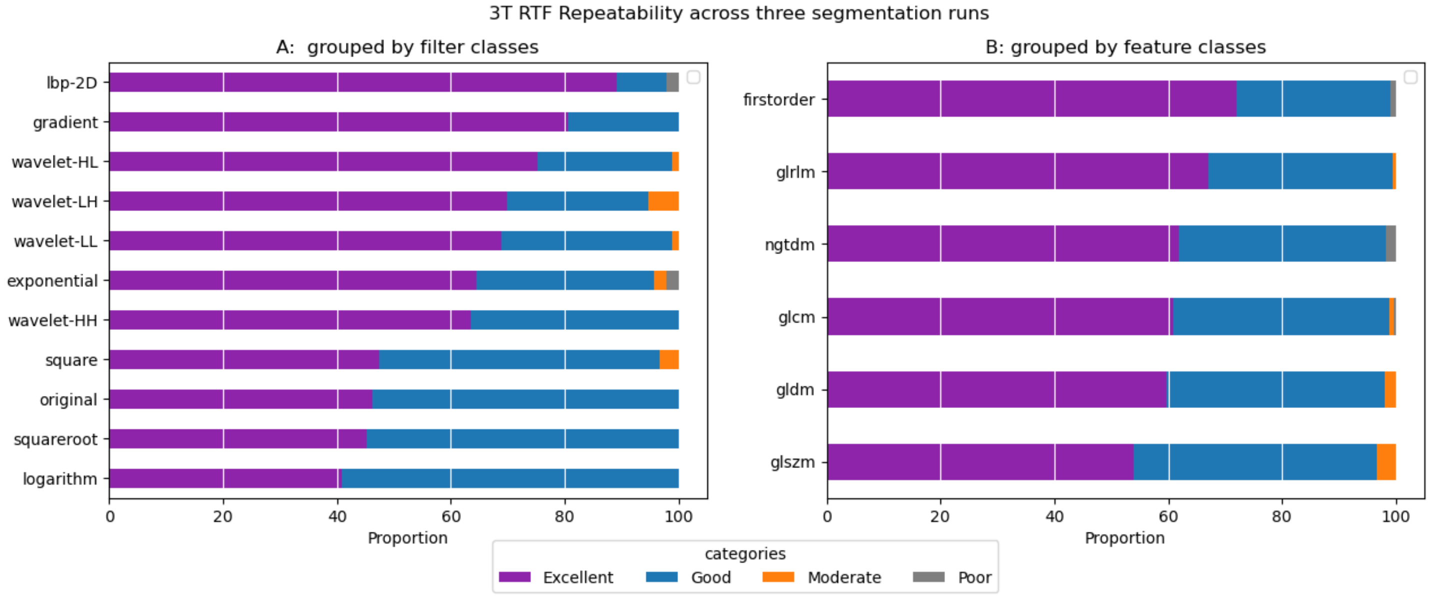

Figure S4 Repeatability of k-averaged measures of RTFs (segmentation mask from run1, run2, and run3) assessed by ICC(3,k). A chart showing the proportion of 3T RTFs from each (A) filter class and (B) feature class that fall in each category of repeatability

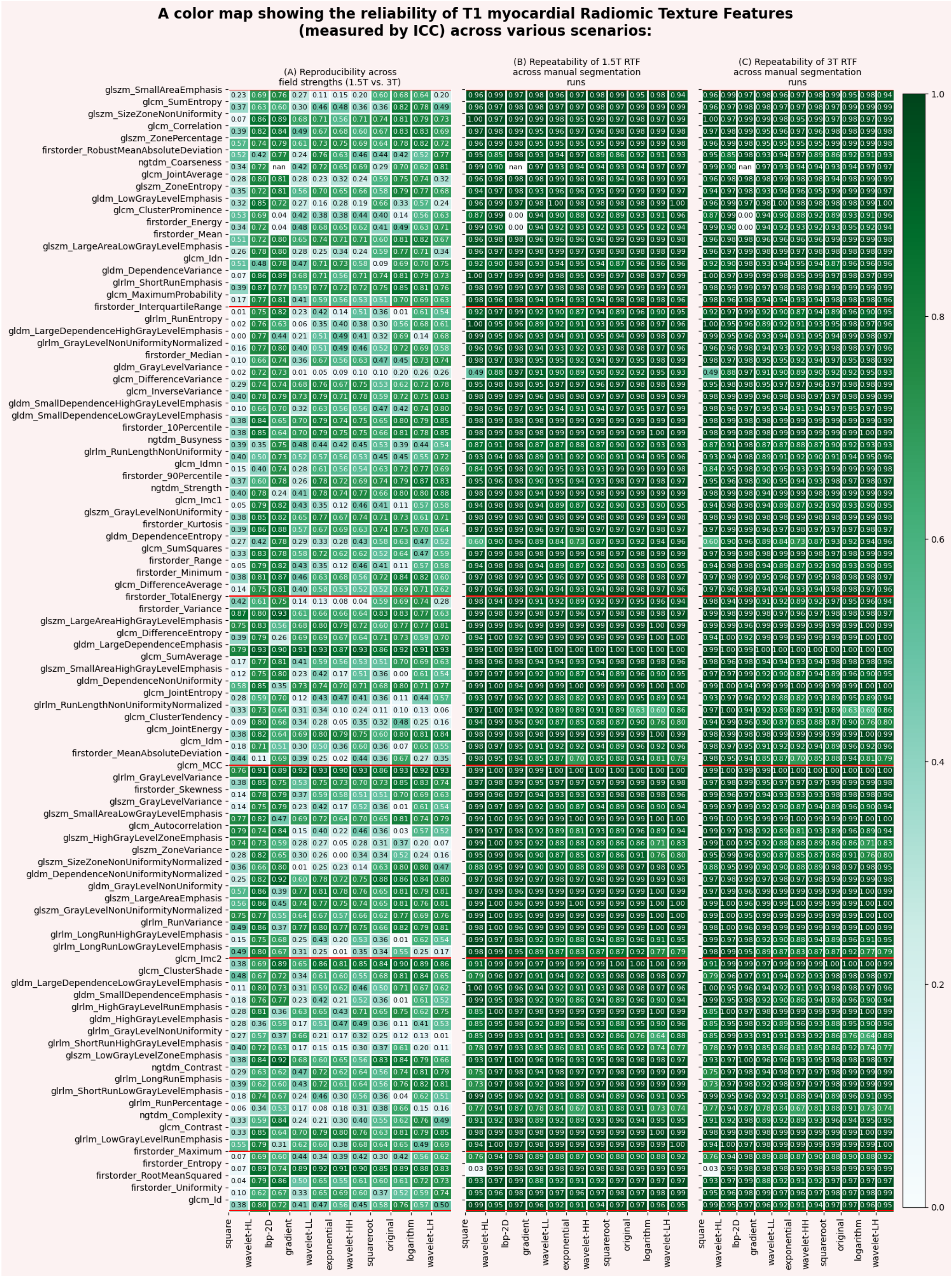

Figure 5 A color grid depicting the reliability of k-averaged measurements for each assessed myocardial radiomic texture feature, with pixels annotated by ICC values. (A) represents the reproducibility across field strengths (1.5T vs. 3T) measured by ICC(2,k). (B) illustrates the repeatability across manual segmentation runs for 1.5T scans, while (C) presents the repeatability for 3T scans, both measured by ICC(3,k).
