# Supplementary material for: Effect of magnetic field strength and segmentation variability on the reproducibility and repeatability of radiomic texture features in cardiovascular magnetic resonance parametric mapping
Source: Int J Cardiovasc Imaging. 2025 Jan 8;41(2):325–37. doi: 10.1007/s10554-024-03312-7 (PMC11811471; doi:10.1007/s10554-024-03312-7)

A color map showing the reliability of T1 myocardial Radiomic Texture Features (measured by ICC) across various scenarios:

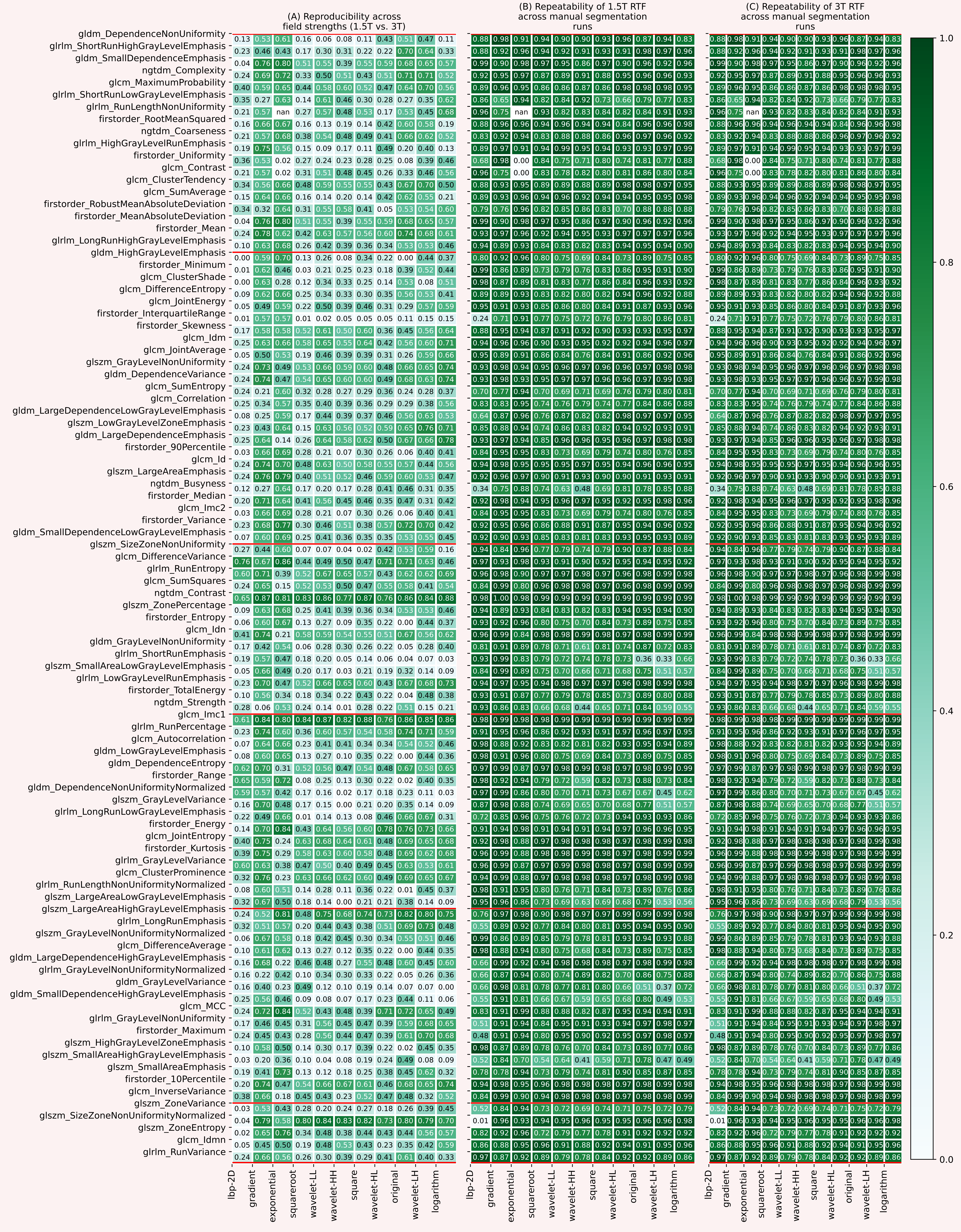

Supplement: Supplementary file 2 — Supplementary file2 (PDF 89 KB) [file 10554_2024_3312_MOESM2_ESM.pdf]
